# HIV-1 Neutralization Profile and Plant-Based Recombinant Expression of Actinohivin, an Env Glycan-Specific Lectin Devoid of T-Cell Mitogenic Activity

**DOI:** 10.1371/journal.pone.0011143

**Published:** 2010-06-15

**Authors:** Nobuyuki Matoba, Adam S. Husk, Brian W. Barnett, Michelle M. Pickel, Charles J. Arntzen, David C. Montefiori, Atsushi Takahashi, Kazunobu Tanno, Satoshi Omura, Huyen Cao, Jason P. Mooney, Carl V. Hanson, Haruo Tanaka

**Affiliations:** 1 Owensboro Cancer Research Program, Owensboro, Kentucky, United States of America; 2 Department of Pharmacology and Toxicology and James Graham Brown Cancer Center, University of Louisville School of Medicine, Louisville, Kentucky, United States of America; 3 Center for Infectious Diseases and Vaccinology, Biodesign Institute, Arizona State University, Tempe, Arizona, United States of America; 4 Department of Surgery, Duke University Medical Center, Durham, North Carolina, United States of America; 5 Faculty of Pharmacy and College of Science and Engineering, Iwaki Meisei University, Fukushima, Japan; 6 KIIM Pharmaceutical Laboratories, Inc., Fukushima, Japan; 7 Kitasato Institute for Lifescience, Kitasato University, Tokyo, Japan; 8 California Department of Public Health, Richmond, California, United States of America; University of California San Francisco, United States of America

## Abstract

The development of a topical microbicide blocking the sexual transmission of HIV-1 is urgently needed to control the global HIV/AIDS pandemic. The actinomycete-derived lectin actinohivin (AH) is highly specific to a cluster of high-mannose-type glycans uniquely found on the viral envelope (Env). Here, we evaluated AH's candidacy toward a microbicide in terms of *in vitro* anti-HIV-1 activity, potential side effects, and recombinant producibility. Two validated assay systems based on human peripheral blood mononuclear cell (hPBMC) infection with primary isolates and TZM-bl cell infection with Env-pseudotyped viruses were employed to characterize AH's anti-HIV-1 activity. In hPMBCs, AH exhibited nanomolar neutralizing activity against primary viruses with diverse cellular tropisms, but did not cause mitogenicity or cytotoxicity that are often associated with other anti-HIV lectins. In the TZM-bl-based assay, AH showed broad anti-HIV-1 activity against clinically-relevant, mucosally transmitting strains of clades B and C. By contrast, clade A viruses showed strong resistance to AH. Correlation analysis suggested that HIV-1′s AH susceptibility is significantly linked to the *N*-glycans at the Env C2 and V4 regions. For recombinant (r)AH expression, we evaluated a tobacco mosaic virus-based system in *Nicotiana benthamiana* plants as a means to facilitate molecular engineering and cost-effective mass production. Biochemical analysis and an Env-mediated syncytium formation assay demonstrated high-level expression of functional rAH within six days. Taken together, our study revealed AH's cross-clade anti-HIV-1 activity, apparent lack of side effects common to lectins, and robust producibility using plant biotechnology. These findings justify further efforts to develop rAH toward a candidate HIV-1 microbicide.

## Introduction

For nearly 30 years, HIV has posed serious global health concerns. Millions of new HIV infections are reported every year worldwide, mainly in developing regions where the availability of antiretroviral drug therapies is extremely limited. As a result, AIDS is among the leading causes of death in these regions [1]. The majority of infections are established via heterosexual transmission and condom use is currently the only available means to directly block this route of infection. As such, the need is urgent for woman-controlled, safe, effective, and inexpensive topical microbicides, until prophylaxis through vaccination becomes globally available [2], [3].

Current candidate microbicides under development encompass chemical and physical agents as well as biologicals, including virion-inactivating agents, entry/fusion inhibitors, reverse transcriptase inhibitors, and others (www.microbicide.org). At this point, it is not known which type of anti-HIV agents will be most effective as topical microbicides; blocking of HIV-1 mucosal transmission may require combinations of multiple agents [4], [5]. Therefore, to broaden the options for different combinations in HIV-1 microbicide development, it is important to expand the candidate portfolio in each category of possible microbicide components.

The envelope (Env) gp120 is heavily glycosylated with *N-*linked glycans (NLGs), which generally account for more than half of the protein's molecular mass [6]. Of these, HMGs represent a major class. Because the Env glycans play critical roles in broad aspects of the viral life cycle ranging from Env folding in host cells to viral transmission and immune escape [7], they constitute an attractive target for entry/fusion inhibitor-based microbicide development.

Lectins have attracted considerable attention in the search for Env glycan-targeting microbicide candidates. Indeed, various naturally occurring lectins have been shown to possess anti-HIV activities. Examples include algae-derived cyanovirin-N (CV-N) and griffithsin (GRFT) as well as plant-derived concanavalin A (Con A) and snowdrop lectin, among others (reviewed in: [7]). More recently, a Jacalin-related lectin isolated from the banana fruit was shown to potently inhibit HIV-1 entry into target cells [8]. Although conceptually not a lectin, the human monoclonal antibody (mAb) 2G12 specifically binds to gp120 HMGs and thereby neutralizes a wide spectrum of HIV-1 strains; thus, it is counted among the very few broadly neutralizing mAbs isolated to date [9], [10].

AH was isolated from the actinomycete strain *Longispora albida* K97-0003^T^ based on the inhibitory activity in a syncytium formation assay [11]. A recent crystallographic analysis revealed that AH is a monomeric protein and possesses three carbohydrate-binding sites [12]. Unlike several other known monosaccharide-specific anti-HIV lectins such as GRFT, Con A, and *Galanthus nivalis* agglutinin [7], AH specifically recognizes a cluster of multiple HMGs via a collaborative action among the protein's three sugar-binding sites [12], [13]. Because clustering HMGs is a unique feature of Env glycans and not usually found on host human proteins [14], AH is hypothesized to be a superior anti-HIV-1 lectin with exquisite specificity to the virus; hence, it may be devoid of unfavorable biological impacts in humans.

In spite of the detailed studies in AH's carbohydrate-binding specificity [12], [13], [15], limited investigation has been reported with regard to the protein's anti-HIV activity in a syncytium formation assay and in a multinuclear-activation-of-galactosidase-indicator (MAGI) assay [15]. Because there is currently no *in vitro* assay that can accurately predict *in vivo* efficacy of a candidate anti-HIV-1 agent, it is important to evaluate the activity of a potential anti-HIV compound in multiple *in vitro* assay systems, preferably, those that can closely simulate the *in vivo* situation and analyze anti-HIV-1 activity in a broad-spectrum of clinically relevant viruses from different clades [16]. Another important factor for a protein-based microbicide candidate is the development of an efficient, cost-effective recombinant expression system that is compatible with extensive preclinical and clinical studies, global distribution, and molecular design for the construction of stronger and/or safer derivatives.

Thus, the primary objectives of our study were to reveal AH's anti-HIV-1 potential in validated *in vitro* neutralization assay systems and to develop a robust expression platform for rAH. To this end, we employed a human peripheral blood mononuclear cell (hPBMC)-based neutralization assay using primary HIV-1 isolates and a reporter gene expressing TZM-bl cell-based neutralization assay using Env-pseudotyped viruses from diverse clades, including clinically relevant C-C chemokine receptor 5-tropic (R5) HIV-1 strains. For recombinant expression of AH, we tested a rapid and robust tobacco mosaic virus (TMV)-based expression system in *Nicotiana benthamiana* plants. Thus, we provide data implicating the feasibilities of AH in terms of its efficacy and production viability. In addition, we performed a preliminary analysis of AH to screen for potential side effects commonly noted with antiviral lectins, i.e., cytotoxicity and mitogenic activity in hPBMCs.

## Methods

### The hPBMC-based primary HIV-1 neutralization assay

The hPBMCs used in the HIV neutralization assay and proliferation analysis described below were purchased from the local blood center (American Red Cross Blood Center, Oakland, CA) as an otherwise-discarded by-product of unsolicited blood donations. No information about identity of the donors was available to the investigators.

The assay was conducted essentially as described in D'Souza et al. [17] and Mascola et al. [18]. Accordingly, the infectious viruses were produced in hPBMCs. The hPBMCs were prepared from buffy coats using Lymphocyte Separation Medium (LSM; Cappel, Aurora, OH). To reduce variation of data from different cell preparations, the cell samples from four different donors were pooled and frozen so that a series of experiments was performed using the same hPBMC pool. An inoculum of 100 50% tissue culture infectious dose (TCID_50_) of HIV-1 was mixed with test samples and incubated for 1 h at 37°C. Next, 3×10^5^ of phytohemagglutinin (PHA)-stimulated hPBMCs were added and incubated for 72 h, and washed to remove residual virus/sample inoculum. After an additional 24-h incubation, cells were lysed and p24 was quantified using a Beckman Coulter (Fullerton, CA) HIV-1 p24 Antigen Assay Research Component Kit. Neutralization was defined as the percent reduction in the amount of p24 detected with the test samples as compared to control. Inhibitory concentrations of 50% (IC_50_) were determined via nonlinear regression analysis using the GraphPad Prism 5 (GraphPad Software, La Jolla, CA). Samples were analyzed in quadruplicate. In each assay, anti-clusters of differentiation antigen 4 (CD4) mAb B4 was used as a positive control [19]. To minimize inter-assay variations of neutralization activity, sample IC_50_s were normalized using B4 IC_50_ values obtained in each assay.

### Cytotoxicity and mitogenic activity in hPBMCs

For cytotoxicity analysis, the CytoTox-ONE™ Homogeneous Membrane Integrity Assay Kit (Promega, Madison, WI) was used to estimate cell viability. hPBMCs were prepared as described above. A test sample was mixed with 2.5×10^5^ hPBMCs and incubated for 6 h at 37°C. Cell viability treated with test samples was calculated by relative lactate dehydrogenase activity in culture medium of that in lysed cell controls.

The proliferative activity of AH in hPBMCs was analyzed by carboxyfluorescein succinimidyl ester (CFSE) staining as described in Elrefaei et al. [20], using the Molecular Probes CellTrace Kit (Invitrogen, Eugene, OR). One million hPBMCs were labeled with 2 µM CFSE and cultured in the presence of a test sample (12.5 µg/ml AH, 1 µg/ml staphylococcal enterotoxin B [SEB, a positive control T cell stimulant [21]], or media alone [negative control]) for 5 days at 37°C in a CO_2_ incubator. Cells were stained with BD Pharmingen anti-human CD4 phycoerythrin (PE), anti-human CD8 peridinin chlorophyll protein with a cyanine dye (PerCP-Cy5.5), and anti-human CD3 allophycocyanin (APC; BD Biosciences, San Jose, CA), and analyzed by an LSR II cell analyzer (BD Biosciences) and FlowJo software (TreeStar, Ashland, OR). Samples were first gated on viable lymphocyte population, and percentage of proliferating cells was determined by measuring the extent of CFSE dilution. The analysis was independently performed three times, using hPBMCs from three different donors.

### The TZM-bl-based Env-pseudotyped HIV-1 neutralization assay

The antiviral activity of AH was assessed based on a reduction in luciferase reporter gene expression after infection of TZM-bl cells with Env-pseudotyped viruses. The assay was performed as described elsewhere [22], except that diethylaminoethyl cellulose dextran was excluded upon infection. Antiviral activity was expressed as an IC_50_ value, which is the sample concentration giving 50% of relative luminescence units (RLUs) compared with those of virus control after subtraction of background RLUs. Env-pseudotyped viruses were prepared by co-transfection of 293T/17 cells with various *env*-expressing plasmids and an *env*-deficient HIV-1 backbone vector (pSG3ΔEnv) and were titrated in TZM-bl cells as previously described [22] to determine TCID_50_. The Genbank accession numbers of the viruses used in the assay are shown in Table 1. The broadly neutralizing mAbs b12, 2G12, 2F5 and 4E10, as well as soluble CD4 were used as positive controls. Two-hundred TCID_50_ of pseudoviruses were used for the neutralization assay. Samples and the virus were mixed and incubated for 1 h at 37°C, to which 10^4^ cells/well of TZM-bl cells were added and incubated for 72 h. Luciferase activity was measured using the Britelite Plus Reagent (PerkinElmer, Waltham, MA).

**Table 1:** Anti-HIV-1 activity of AH against Env-pseudotyped viruses in TZM-bl cells.

		Genbank Accession No.		IC_50_ (µg/ml)*	
Virus	Subtype		Tropism	2G12	AH
MN.3	B	AY669737.1	X4	ND	0.04
SF162.LS	B	EU123924.1	R5	0.7	>25
W61D 7.12	B	AY973156.1	R5/X4	ND	9.9
Bal.26	B	DQ318211.1	R5	0.9	1.5
SS1196.1	B	AY835442.1	R5	10.8	0.01
Bx08.16	B	GQ855765.1	R5	5.4	2.3
BZ167.12	B	GQ855764.1	R5/X4	ND	2.9
6535.3	B	AY835438.1	R5	2	>25
QH0692.42	B	AY835439.1	R5	2.8	13.8
TRO.11	B	AY835445.1	R5	0.4	6.7
RHPA.7	B	AY835447.1	R5	>50	15.1
TV1.21	C	AF391231.1	R5	1.7	0.3
92BR025.9	C	U15121.1	R5	1.2	1.8
MW965.26	C	U08455.1	R5	>25	2.5
ZM214M.PL15	C	DQ388516.1	R5	>50	21.2
ZM109F.PB4	C	AY424138.2	R5	>50	>25
Q461.e2	A	AF407156.1	R5	>50	>25
Q259.d2.17	A	AF407152.1	R5	>50	>25
Q842.d12	A	AF407160.1	R5	>50	>25
Q168.a2	A	AF407148.1	R5	>50	>25

*IC_50_s exceeding the highest concentrations of samples tested in the assay are expressed as >25 or >50 µg/ml. ND: not determined.

### Correlation analysis

Env potential NLG sites were determined based on sequons (Asn-X-Thr/Ser-Y, where X and Y are any aa except for Pro) using in Env sequences of viruses tested in the TZM-bl-based assay (provided in Genbank) and the online tool N-Glycosite at Los Alamos HIV Database (http://www.hiv.lanl.gov/content/sequence/GLYCOSITE/glycosite) [23]. The correlation between the IC_50_s and the number of sequons at whole or the selected Env region was analyzed using the non-parametric Spearman's correlation coefficient in the GraphPad Prism 5 software. To allow this analysis using all the viruses tested in the TZM-bl-based assay, any IC_50_ values greater than the highest concentration tested were arbitrary approximated to the next two-fold dilution step (i.e., 50 µg/ml for experimental IC_50_s >25 µg/ml).

### rAH expression in *N. benthamiana*

A “deconstructed” TMV replicon system (magnICON; Icon Genetics GmbH, Halle/Saale, Germany) was used [24], [25] to express rAH in *N. benthamiana*. The native *ath* gene (Genbank accession no. AB032371) was sub-cloned into the gene expression module pICH11599 using Nco I and Sac I restriction sites to form pNM86. The three component plasmids (pNM86, pICH20111, and pICH14011) were each transferred into the *Agrobacterium tumefaciens* strain GV3101 by electroporation. Bacteria were resuspended in an infiltration buffer (10 mM 2-(*N*-morpholino)ethanesulphonic acid [MES], 10 mM MgSO_4_, pH 5.5). Equal portions of the three bacteria were then mixed to give an optical density at 600 nm (OD600) of 0.1. *N. benthamiana* plants were grown at 27°C and 55–65% humidity for 4 weeks under an 18 h-light/6 h-dark cycle. Forty-eight h before inoculation, plants were moved to an incubator set at 22°C and 55–65% humidity with the same lighting cycle. The bacteria suspension was infiltrated into leaves by application of a vacuum for 2 min at 25 inches Hg using a vacuum pump. After infiltration, plants were placed in the incubator set at 22°C and kept in darkness for 16 h. Subsequently, the incubator was set to a normal 18 h-light/6 h-dark cycle. At 4 to 6 days post infiltration (dpi), infected leaves were harvested and examined for rAH expression as described below.

### Detection of rAH

Expression of rAH was analyzed by sodium dodecyl sulfate-polyacrylamide gel electrophoresis (SDS-PAGE) and western blotting. Leaf materials were extracted with 5 v/w of SDS extraction buffer (50 mM Tris-HCl [pH 6.8], 2% SDS, 0.003% bromophenol blue, 10% glycerol). After electrophoresis, gels were stained with Coomassie Brilliant Blue, or the resolved proteins were electro-transferred to a poly (vinylidene difluoride) membrane. Blots were probed with rabbit anti-AH antiserum (1∶5,000) followed by horseradish peroxidase (HRP)-conjugated goat anti-rabbit immunoglobulin (Ig)G (1∶10,000; Santa Cruz Biotechnology, Santa Cruz, CA), which were then detected using a chemiluminescence luminol reagent (Santa Cruz Biotechnology).

The amounts of rAH in extract were quantified by gp120-captured enzyme-linked immunosorbent assay (gp120-ELISA). ELISA plates were coated with 0.3 µg/ml of HIV-1 gp120 CM Env protein (National Institute of Health AIDS Research and Reference Reagent Program [NIH ARRRP], Washington, D.C.) and blocked with blocking buffer (phosphate-buffered saline [PBS], pH 7.2, 0.05% Tween-20, 5% [w/v] non-fat dry milk). Serially diluted extract samples were applied onto the plates and incubated for 1 hr at 37°C. The gp120-bound rAH was detected by rabbit anti-AH antiserum (1∶3,000) followed by HRP-conjugated goat anti-rabbit IgG (1∶10,000). A tetramethylbenzidine substrate (BioFX Laboratories, Owings Mills, MD) was used for detection; absorbance at 450 nm was measured with a plate reader (Beckman Coulter). A standard curve was created for each plate using standard AH (5–300 ng/ml; purified from original actinomycete culture), with which the amounts of rAH in samples were calculated using the SoftMax Pro software (Molecular Devices, Sunnyvale, CA).

### Syncytium Formation Assay

The assay was performed as previously described, using HeLa/env/tat and HeLa/CD4/lacZ cell lines [26]. To prepare leaf extract samples for analysis, leaf materials were homogenized in 5 v/w of extraction buffer 1 (PBS, pH 7.4, 20 mM ascorbic acid, 10 mM sodium metabisulfite) and centrifuged. Most (>99%) of the total gp120-binding rAH was retained in the insoluble pellet fraction. The pellet was re-extracted with extraction buffer 2 (50 mM glycine, pH 2.5, 20 mM ascorbic acid, 10 mM sodium metabisulfite, 6 M guanidine HCl). The extract was clarified by centrifugation and dialyzed against 50 mM glycine (pH 2.5). The dialyzed sample was centrifuged to remove insoluble materials and concentrated using a centrifugal concentrator (3.5 kDa molecular weight cut-off). The amount of rAH in each fraction was quantified by gp120-ELISA, as described above. Typically, in the dialyzed sample we obtained ∼25 µg of rAH from one g of leaf material.

For a quantitative assay, serially diluted samples and 9×10^3^ cells/well of HeLa/env/tat and HeLa/CD4/LacZ cells were mixed in GIBCO Dulbecco's modified Eagle's medium (DMEM; Invitrogen, Carlsbad, CA) containing 10% fetal bovine serum, 1% penicillin/streptomycin and 100 µg/ml kanamycin on a 96-well plate and incubated for 18 h at 37°C in a CO_2_ incubator. Cells were washed and lysed with 0.05% Triton X-100. To quantify syncytia, a developing reagent (60 mM Na_2_HPO_4_, 40 mM NaH_2_PO_4_, 10 mM KCl, 1 mM MgSO_4_, 50 mM 2-mercaptoethanol, 0.8 mg/mL ortho-Nitrophenyl-β-galactoside) was added and incubated at room temperature for 2 to 4 h. After stopping the reaction with 2 M Na_2_CO_3_, the OD_450_ was read.

For staining of syncytia, HeLa/env/tat and HeLa/CD4/LacZ (10^5^ cells each/well) with or without a test sample were incubated on an 8-well microscope slide (Nalge Nunc International, Rochester, NY) for 18 h at 37°C. Cells were washed with PBS, and fixed with 4% paraformaldehyde. Syncytia were stained with 5-bromo-4-chloro-3-indolyl-β-D-galactopyranoside (X-gal) staining solution (25 ml PBS, pH 7.4, 2 µg/ml MgCl_2_, 1.64 mg/ml potassium ferricyanide, 2.12 mg/ml potassium ferrocyanide, 1 mg/ml X-gal) for 18 h at 37°C and observed under an optical microscope.

## Results

### AH inhibits hPBMC infection by primary HIV-1 isolates

**Figure 1:**
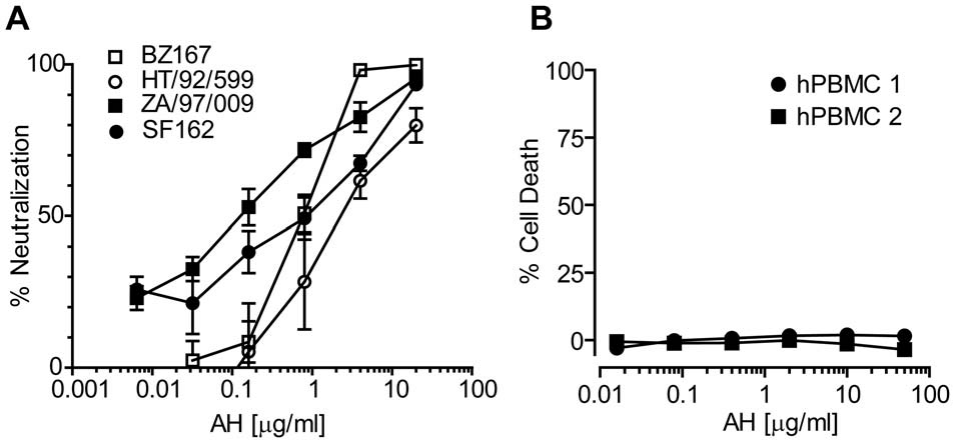
Neutralization of primary HIV-1 isolates in hPBMCs. (A) The hPBMC-based neutralization assay. The reduction of p24 gag protein production in hPBMCs was measured as an endpoint. hPBMCs (pooled from four different donors) were infected with R5 B clade (SF162), R5 C clade (ZA/97/009), X4 B clade (HT/92/599), or R5X4 B clade (BZ167) in the presence of various concentrations of AH. Samples were analyzed in quadruplicate.Results are expressed as mean ± SEM. See Materials and Methods for assay details. Dose-dependent neutralization was observed for all viruses. (B) Cytotoxicity analysis. Potential toxicity of AH in hPBMCs was analyzed based on the LDH activity in the culture medium after incubation of hPBMCs with various concentrations of AH. Analysis was performed in quadruplicate in two different pools of hPBMCs. Results are expressed as mean ± SEM. The lack of hPMBC cytotoxicity was demonstrated for up to 50 µg/ml of AH.

Given that clustering HMGs is a conserved feature of the HIV-1 Env, broad anti-HIV-1 activity has been suggested for the HMG-specific lectin AH. To prove this concept in an assay system simulating the physiological conditions, we performed a hPBMC-based neutralization assay using primary HIV-1 isolates [17], [18]. Viruses with diverse cellular tropisms were tested, i.e., an R5 B clade (SF162), an R5 C clade (ZA/97/009), a chemokine (C-X-C motif) receptor 4-tropic (X4) B clade (HT/92/599), and a dual-tropic R5X4 B clade (BZ167). The p24 production in the cells was quantified to monitor HIV-1 infectivity. As shown in Figure 1A, AH neutralized all the viruses with IC_50_s within the nanomolar range (1 µg/ml of AH corresponds to 80 nM). Among the four primary isolates tested, the R5-type C clade virus showed the highest susceptibility to AH (IC_50_ = 0.12 µg/ml or 9.6 nM and IC_90_ = 12.10 µg/ml or 968 nM) whereas the X4 B clade virus was relatively resistant (IC_50_ = 2.68 µg/ml or 224 nM and IC_90_ = 29.67 µg/ml or 2,370 nM). These results are congruent with the previous findings in a surrogate MAGI assay, where AH exhibited antiviral activity at the nanomolar dose range (IC_50_s = 2–110 nM) against three laboratory-adapted T- and M-tropic strains (IIIB, NL4-3, and JR-CFS) and one T-tropic primary isolate (O18A) [15]. Meanwhile, AH did not show any cytotoxicity in hPBMCs at the highest concentration tested (50 µg/ml or 4 µM), indicating that the reduction of p24 production in the assay was not due to cytotoxicity (Fig. 1B). Taken together, these results demonstrated that AH can inhibit the infection of hPBMCs by primary HIV-1 strains irrespective of cellular tropism, which directly suggests the possibility of the protein's *in vivo* efficacy.

### AH does not induce hPBMC proliferation at a dose exerting strong anti-HIV-1 activity

**Figure 2:**
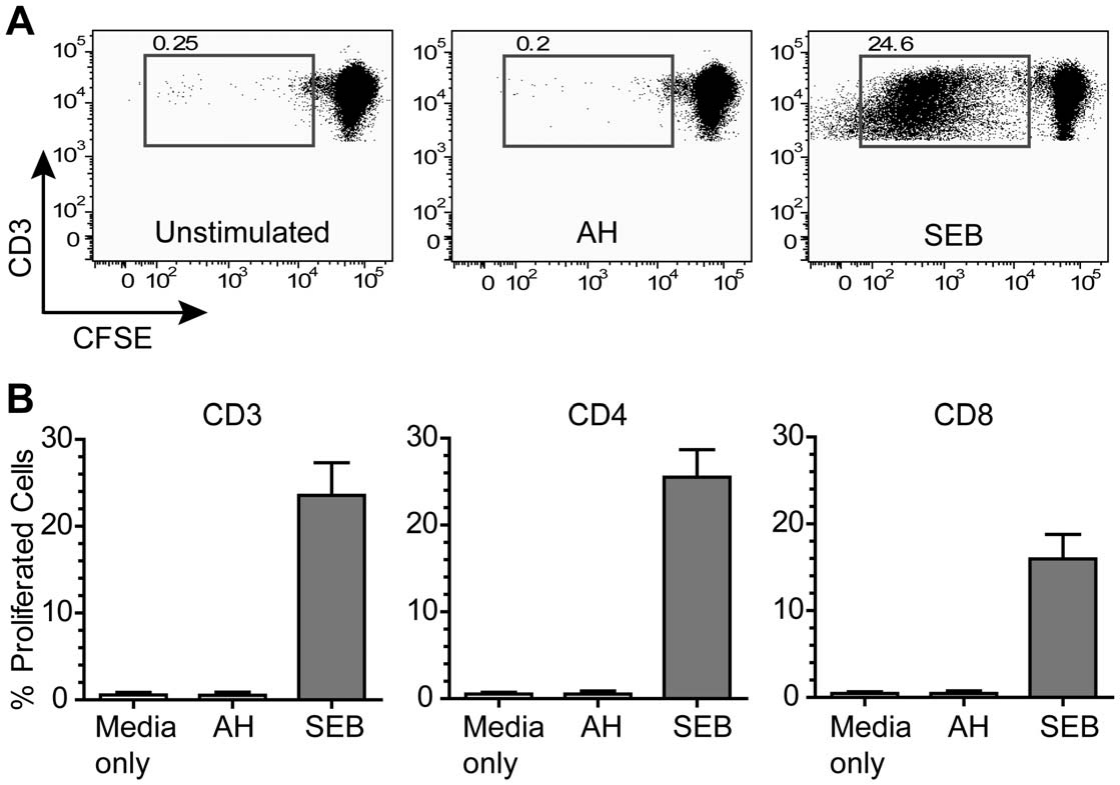
Analysis of mitogenic activity in hPBMCs. Proliferation of human CD3^+^ lymphocytes in hPBMCs was analyzed by flow cytometry. CFSE-stained hPBMCs were incubated for 5 days with 12.5 µg/ml AH, 1 µg/ml SEB (positive control; a potent T cell stimulator [21], [58]), or media alone. Samples were first gated on a viable T cell population, and the percentage of proliferating cells was determined based on the extent of CFSE dilution. (A) Representative plots from one of three experiments are shown for CD3^+^ cells. The number in each panel (above the boxed plot population) indicates the percentage of proliferated cells. (B) Results from 3 independent experiments using different hPBMCs are shown. In addition to CD3^+^ cells, CD4^+^ and CD8^+^ cells were analyzed to further dissect proliferative potential. Results are expressed as mean ± SD. AH did not show any mitogenic activity in any cell types tested.

Many lectins are known to exhibit various biological effects on human cells via their specific carbohydrate binding. For example, Con A and PHA are well-known mitogens in human T cells [27]. The banana-derived lectin entry inhibitor BanLec effectively proliferated CD3^+^, CD4^+^ and CD8^+^ populations in hPBMCs at 2 µg/ml (corresponding to ∼67 nM as a natural dimer form) [28]. Furthermore, strong mitogenic activity and cytotoxicity in hPBMCs was observed for CV-N, which has been one of the most studied HMG-specific anti-HIV-1 lectins to date [29], [30]. Because hPBMC mitogenicity may lead to the disastrous side effect of *enhanced* HIV-1 infectivity in these cells [29], it is imperative to demonstrate that the anti-HIV-1 effect of a candidate microbicide is not accompanied by mitogenic activity in these cells. Therefore, we tested the proliferation potential of hPBMCs upon exposure to AH. As shown in Figure 2, AH at 1 µM (or 12.5 µg/ml) did not show any sign of proliferative activity either in a whole CD3^+^ T cell population or in CD4^+^ or CD8^+^ cells after prolonged incubation (for 5 days) with AH (Fig. 2). These results are in sharp contrast to the protein's nanomolar anti-HIV-1 activity in the hPBMC-based neutralization assay, where the same dose exerted a prominent HIV-1 neutralization effect against primary isolates (Fig. 1A). Coupled with the data showing AH's lack of cytotoxicity up to 4 µM (see Fig. 1B), it is suggested that AH anti-HIV-1 activity is not associated with some of the major common side effects noted with other antiviral lectins.

### Broad-spectrum anti-HIV-1 activity of AH against R5-type viruses

To extend our examination on the breadth of AH's anti-HIV-1 activity, we performed a reporter gene expression TZM-bl-based neutralization assay using Env-pseudotyped viruses [22]. We included multiple R5 A, B and C clade viruses in the analysis because: 1) R5 viruses are transmitted predominantly during the course of HIV-1 transmission [31], [32] and are therefore particularly important from an HIV-1 microbicide standpoint; and 2) these three clades represent more than 70% of the viruses currently circulating in the world [33], [34]. Table 1 shows IC_50_s of AH against clade A, B, and C isolates of AH and the HMG-specific broadly neutralizing mAb 2G12. AH showed broad anti-HIV-1 activity that was similar overall to that of 2G12, aside from a few exceptions. Among the viruses tested, ZM109F.PB4 (R5 C clade) and all the four A clade viruses showed IC_50_s of over 25 µg/ml for both AH and 2G12. These results may indicate that clade A viruses are generally highly resistant to the two HMG-specific anti-HIV proteins compared to other subtypes. By contrast, the notable difference between AH and 2G12 was seen with other viruses. SF162 (B clade R5) and 6535.3 (B clade R5) showed complete resistance to AH (IC_50_s >25 µg/ml) but not to 2G12, while RHPA4259.7 (B clade R5), MW965.26 (C clade R5), and ZM214M.PL15 (C clade R5) were susceptible to AH but resistant to 2G12. These results indicate that the Env glycan target and/or anti-HIV mode are different between these two proteins. Of interest is the fact that SF162, which showed relatively high susceptibility to AH in the hPBMC-based assay (IC_50_ = 0.48 µg/ml, Fig. 1), was highly resistant to this protein in the TZM-bl-based neutralization assay. On the other hand, the dual-tropic B clade BZ167, another virus strain used in both assay formats, showed similar sensitivity to AH (IC_50_s at 0.8 µg/ml in the hPBMC-based assay vs. 2.9 µg/ml in the TZM-bl-based assay).

**Figure 3:**
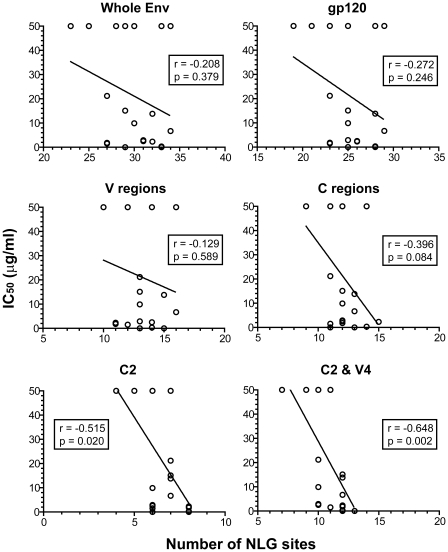
Correlation between the number of NLG sequons and AH susceptibility in Env-pseudotyped viruses. For each virus tested in the TZM-bl-based assay, the number of NLG sites (i.e., sequons) in the indicated Env region was plotted against the IC_50_. The IC_50_ values of >25 µg/ml were approximated to 50 µg/ml. Correlation was analyzed by the non-parametric Spearman's correlation coefficient. The correlation coefficient (r) and *p* values are shown in the box in each graph. A *p* value of less than 0.05 is regarded as statistically significant. Linear regression analysis was used to display a best fit line to the data.

**Figure 4:**
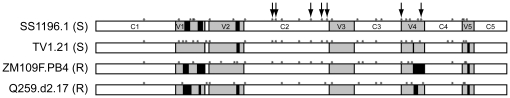
Schematic representation of Env and potential NLG positions in selected AH-susceptible and -resistant viruses. Constant regions (C1–C5; shown in white) and variable regions (V1–V5; shown in grey) are shown for the two most susceptible viruses (SS1196.1 and TV1.21) and two highly resistant viruses (ZM109F.PB4 and Q259.d2.17) to AH, according to the results shown in Table 1. Black boxes indicate deletions in the corresponding regions. Asterisks represent the position of sequons. Potential targets of AH within the C2 and V4 regions (those present in both the susceptible viruses but absent in either of the resistant strains) are indicated by arrows.

To partly discern whether AH's anti-HIV-1 effect is associated with a particular Env NLG pattern, the IC_50_s obtained in the TZM-bl-based neutralization assay were plotted against the number of potential *N-*glycosylation sites on Env (Fig. 3). There was no clear correlation between AH-susceptibility and the number of sequons on whole Env or V regions. On the contrary, a relative correlation was observed between AH susceptibility and sequons at entire C regions (Spearman correlation coefficient [r]  = −0.40, *p* = 0.084). Site-specific analysis revealed that sequons at the C2 segment alone have a significant correlation (r = −0.51, *p* = 0.020). Notably, this correlation became even more apparent when sequons at the C2 and V4 regions were combined (r = −0.65, *p* = 0.002), even though V4 sequons alone were not strong enough to show a significant correlation with AH-susceptibility (*p* = 0.176). Throughout the Envs from all the viruses analyzed, there was no significant change in the number of sequons in C1, C4, C5, V3, or gp41. Relatively high degrees of numerical variations were observed in V1/V2, V5, and C3 regions, but no correlation with AH susceptibility was detected in these regions (data not shown). Taken together, these results suggest that NLGs at C2 and V4 may constitute the sole and/or critical targets of AH. In Figure 4, we compared Env NLG positions of the highly susceptible viruses (R5 B clade SS1196.1 and R5 C clade TV1.21) and resistant strains (R5 C clade ZM109F.PB4 and R5 A clade Q259.d2.17). It was suggested that clustering NLGs at the middle and C terminal region of C2 as well as in V4 may play an important role in AH's anti-HIV-1 activity.

For a comparison, we also performed the same analysis with 2G12. It was revealed that 2G12 susceptibility showed a statistically significant correlation with the total number of sequons located at C2, C3 and V4 (r = −0.52, *p* = 0.034) and with V4 sequons alone (r = −0.53, *p* = 0.028), but not with any other Env regions (data not shown). These results correspond well with previous mutagenesis and crystallographic studies that have defined the Env NLGs targeted by 2G12 [10], [35], thus arguing for the present approach to predict AH's potential binding target(s).

### Expression of rAH in *N. benthamiana* using a modified TMV vector

**Figure 5:**
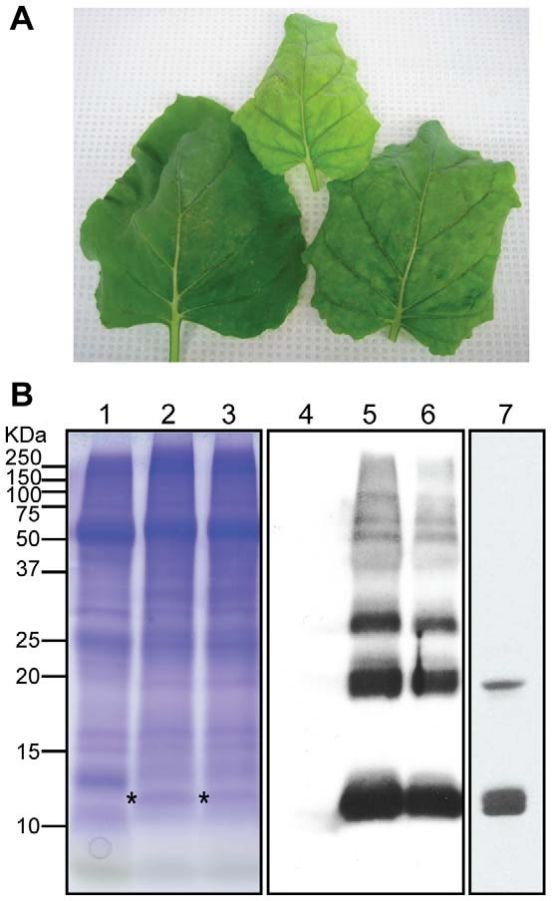
Expression of rAH in *N. benthamiana*. (A) Representative TMV vector-infiltrated *N. benthamiana* leaves at 6 dpi. The leaves show only a minor level of tissue necrosis. (B) Electrophoretic analysis of rAH expression in leaf extract. Coomassie-stained SDS-PAGE gel (Lanes 1–3) and western blot analysis (Lanes 4–7) are shown. Total leaf proteins were extracted in a buffer containing 2% SDS and separated under reducing conditions. Lanes 1 and 4: non-infiltrated control leaf extract; lanes 2, 3, 5, and 6: vector-infiltrated leaf extracts made from two independent infiltration events; and lane 7: the dialyzed aqueous fraction of infiltrated leaf proteins prepared by 6 M guanidine buffer extraction. Asterisks indicate the bands corresponding to rAH (Mw: 12.5 kDa). Efficient extraction of gp120-binding rAH required a buffer containing a chaotropic agent such as SDS or guanidine HCl. The fraction contains apparent multimers of rAH (Lanes 5 and 6). These aggregates are largely absent after dialysis, while the monomer protein stays soluble (Lane 7). See text for detail.

Next, we focused on the development of an efficient rAH expression system to facilitate the production and molecular engineering of the protein. For this purpose, a modified TMV-based expression system was employed [24], [25]. Under our normal plant growth conditions at 27°C, severe necrosis emerged in the infiltrated leaves at 4 to 5 dpi. However, the necrosis was significantly reduced by lowering the plant cultivation temperature by 5°C, i.e., to 22°C (Fig. 5A). The expression level of rAH did not change by this modification, according to gp120-ELISA (data not shown). As shown in Figure 5B, the expression of rAH (Mw: 12.5 KDa) was clearly detected at 5 dpi on an SDS-PAGE gel. Western blot analysis using polyclonal anti-AH Abs further confirmed the expression (Fig. 5B). Interestingly, however, apparent oligomers (dimer, trimer, etc) and aggregates were detected along with the monomeric 12.5 kDa protein (Fig. 5B). No oligomer formation has previously been noted for the original actinomycete-derived AH [11], [12], although an apparent dimer was observed in *E. coli*-expressed rAH [36] and a minor amount of dimers exists in a purified actinomycete-derived AH sample (Matoba et al., unpublished observation). Therefore, the oligomer/aggregate formation is unique to the TMV-based expression. The gp120-ELISA of the leaf extract revealed that the expression of rAH peaked at 6 dpi, with expression levels ranging from 20 to 120 mg per kg of fresh leaf material (data not shown), which generally agrees with the intensity of the band in SDS-PAGE in Figure 5B.

**Figure 6:**
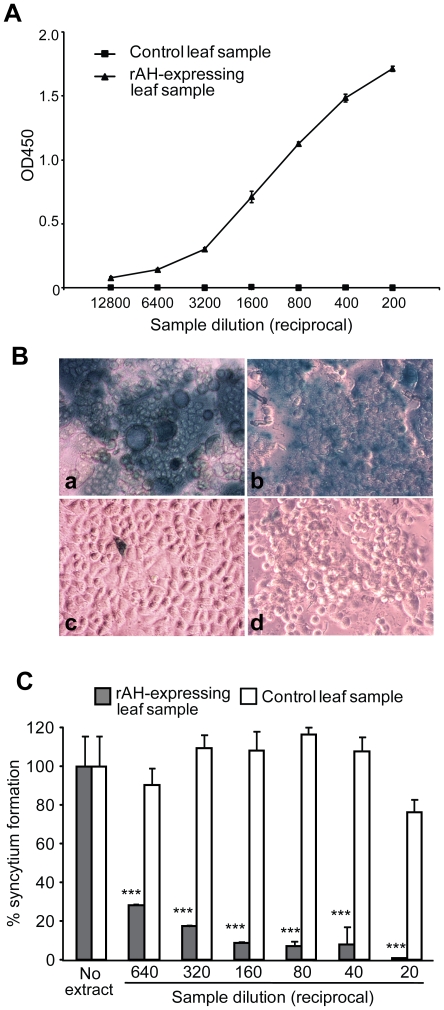
Activity analysis of *N. benthamiana*-expressed rAH. (A) The gp120-binding capacity of rAH expressed in *N. benthamiana* was evaluated by gp120-ELISA using AH-expressing and control (uninfiltrated) leaf extracts prepared as described in Materials and Methods. Results of a representative sample obtained from a ∼2 g batch of leaf materials are shown. Data points are the means ± SD of triplicate analysis. This analysis was also used to determine the quantity of rAH in the extract based on a standard curve created by actinomycete-derived purified AH. (B) X-gal staining of syncytia formation by HeLa/Env/tat and HeLa/CD4/LacZ. The two cell lines were incubated for 18 h at 37°C with: **a**, media alone; **b**, 1/20-diluted non-infiltrated control leaf sample; **c**, 1 µM actinomycete-derived AH; and **d**, 1/20-diluted rAH-expressing leaf sample (containing approximately 0.32 µM, or 4.0 µg/ml, of rAH, as determined by gp120-ELISA). (C) Quantitative analysis of syncytia formation by HeLa/Env/tat and HeLa/CD4/LacZ. The two cell lines were incubated for 18 h at 37°C with serially diluted control or rAH-expressing leaf samples. See Materials and Methods for the assay detail. Differences between the samples at each dilution point were evaluated by two-way analysis of variance followed by Bonferroni posttests (*** *p*<0.001).

The efficient recovery of the gp120-binding fraction of rAH from the leaf required an acidic extraction buffer containing a chaotropic agent such as urea or guanidine HCl. After removing the denaturant by dialysis, however, rAH remained soluble and was largely devoid of high-order oligomers (Fig. 5B, lane 7). The soluble plant-expressed rAH strongly bound to gp120 (Fig. 6A). To further demonstrate plant-expressed rAH's functional integrity, we performed a reporter gene expression syncytium formation assay using cells expressing human CD4 and HIV-1 Env. As seen in Figure 6B, the X-gal staining of the cells clearly shows that plant-expressed rAH (Fig. 6B–d), but not a reference control prepared from a non-infiltrated leaf sample (Fig. 6B–b), inhibited the formation of syncytia. No inhibition was observed with a sample from GFP-expressing leaves obtained by the same TMV-based vector (data not shown). This observation was further confirmed by a quantitative analysis. Thus, plant-expressed rAH dose-dependently and significantly inhibited the syncytia compared to a control leaf sample (Fig. 6C). According to the gp120-ELISA in Fig. 6A, a 1/20-diluted rAH sample contained 322 nM of the protein (corresponding to the far-right bar in Fig. 6C). The results were in good agreement with the syncytium-inhibitory pattern of the original AH sample (data not shown), indicating the integrity of the plant-expressed protein. Taken together, these results demonstrate that plants can serve as a new recombinant expression platform for AH and that plant-expressed rAH is functionally active.

## Discussion

For nearly thirty years, HIV/AIDS has posed a serious global public health concern. In the most pandemic sub-Saharan African countries, heterosexual exposure constitutes the major route of HIV-1 transmission, with half of the new infections occurring among women [1]. Although condom use and male circumcision can significantly reduce the chance of HIV-1 sexual transmission, there is no direct countermeasure currently available at women's disposal [1]. Therefore, the development of a woman-controlled microbicide is urgently needed for the control of the pandemic. Three key factors are proposed for candidate microbicides to move from the preclinical to clinical phase, i.e., efficacy, safety, and economical viability [3]. We have therefore initiated our investigation on the feasibility of AH in these aspects.

AH is an actinomycete-derived anti-HIV protein that has high specificity to HMG clusters on Envs. Because HMGs constitute a conserved major fraction of Env NLGs, the protein possesses high potential as an anti-HIV agent. In the two *in vitro* HIV-1 neutralization assays performed here, we obtained several key advances in understanding the anti-HIV-1 activity profile of AH.

First, AH's anti-HIV-1 effect was demonstrated in a hPBMC-based assay using primary isolates and in a TZM-bl-based assay using Env-pseudotyped viruses of clinically-relevant strains from major clades. These two *in vitro* assay formats are currently recommended for evaluating the anti-HIV-1 activity of neutralizing Abs, hence entry/fusion inhibitors, to best predict their *in vivo* antiviral efficacies [37]. In both assay systems AH exhibited clear anti-HIV-1 effects against many clinically relevant viruses, including those of the mucosally transmitted R5-type. In addition, the activity dose ranges of AH were similar between the two systems overall, which are also consistent with previous results in another cell-line based surrogate neutralization assay system (IC_50_s at the nanomolar range; [15]). Taken together, it is strongly argued that AH is a broadly neutralizing anti-HIV-1 lectin that could potentially blunt mucosal HIV-1 transmission of primary R5 viruses. Further studies are required to predict the protein's activity upon microbicide use. For example, it is critical to test the anti-HIV-1 effect in *in vitro* studies more closely simulating the physiological conditions in the vagina, e.g., in the presence of seminal plasma and under a pH transition that takes place during the coital event [38]. Sustained efficacy under these conditions, along with safety (as discussed below), are prerequisites for any microbicide candidate.

While AH's anti-HIV-1 effects in the hPBMC-based- and TZM-bl-based neutralization assays fell into a similar range, there was a significant difference in the sensitivity of the B clade SF162 to AH between the two systems (IC_50_ was 0.5 µg/ml in the former and >25 µg/ml in the latter; see Fig. 1 and Table 1). Multiple studies have reported similar discrepancies in inhibitory patterns of neutralizing Abs between the two assay systems [16], [37]. In our case, the conflicting result may be a reflection of the phenotypic difference in viruses produced in these assays, i.e., the primary isolate produced in hPBMCs versus the Env-pseudotyped virus produced in 293T cells. It has been shown that the *N-*glycan structure of viral glycoproteins can vary depending on the cell type in which the virus is produced [14], [39], [40]. For example, HIV-1 gp120 s produced in human PBMCs were shown to have more HMGs than those produced in monocyte-derived macrophages [14]. Because AH targets Env HMGs, it is conceivable that viruses harboring more HMGs on their Envs would be more susceptible to the protein. Thus, although there is no direct information as to the glycan profiles of SF162 Envs produced in hPBMCs and 293T cells, the higher AH sensitivity of the hPBMC-produced virus may be explained by the higher number of HMGs on the Env. Alternatively, a potential difference in the Env density on the viruses used in the two different systems may account for the discrepancy in viral AH sensitivity, as has been reported [41], [42]. In any event, the above may imply that a future efficacy study of AH or other relevant anti-HIV-1 agents should include testing of viruses present in semen [43], [44].

Second, we uncovered the breadth of AH's anti-HIV-1 effect in the TZM-bl-based neutralization assay. It was revealed that AH possesses anti-HIV-1 activity against many clinically relevant R5 viruses, having the neutralization breadth similar to that of the HMG-targeting broadly neutralizing mAb 2G12. This is not surprising because both 2G12 and AH recognize the Manα1–2Man moiety of HMGs [9], [10], [12], [35]. However, a few discrepancies in the neutralization patterns between 2G12 and AH indicate that their target HMGs are not identical (discussed later). AH may generally be more effective against R5 C clade viruses than 2G12, as the former neutralized four viruses while the latter inhibited only two out of the five viruses tested at lower than 25 µg/ml (Table 1). This points to an advantage of AH because the C clade is highly prevalent in epidemic regions where over 80% of total global HIV-1 infections occur [33], [34], although analysis must be expanded to validate this concept. On the other hand, both AH and 2G12 showed lack of efficacy to all the four A clade viruses. Interestingly, relative resistance of A clade viruses has previously been observed for another potent mannose-specific anti-HIV-1 lectin, GRFT [45]. These results may indicate the general resistance of A clade viruses to HMG-targeting HIV-1 inhibitors and imply a potential weakness for this class of anti-HIV agent. Alternatively, this warrants investigation of more in depth clade-specific analysis and identification of a different type of anti-HIV agent that can supplement AH or other HMG-targeting HIV-1 inhibitors toward an effective combination microbicide strategy.

Third, correlation analysis between AH susceptibility and Env NLG profiles suggested that several NLGs might be targeted by AH. These glycans tend to cluster at the middle and at the C-terminal region of C2 and at V4 (Fig. 4). A recent crystallographic analysis indicated that AH possesses three carbohydrate-binding sites and that each of these appeared to accommodate a couple of Manα1–2Man moieties from the D1 and D3 arm of a single HMG [12]. Our results suggest that NLGs at the C2 and V4 regions may comprise a set of HMGs recognized by AH's three sugar binding sites. This notion is supported in part by the previous studies showing that NLGs at these locations tend to contain more HMGs than other regions [46], [47], [48]. Obviously, further studies are needed to demonstrate AH's target Env NLGs, e.g., crystallography of an AH-gp120 complex, and analysis of escape and mutant viruses displaying modified Env NLG patterns. Such studies will also help dissect the molecule's anti-HIV-1 mechanism to aid the development of potent AH analogues and are ongoing in our laboratories.

AH is similar to CV-N in that both lectins recognize the Manα1–2Man structure of HMGs and thereby exert anti-HIV-1 activity, although the former relies on high-avidity interaction with multiple HMGs while the latter is specific to a single HMG [12], [49], [50]. CV-N is a leading lectin-based microbicide candidate that has shown promising results in macaque challenge studies [51], [52] and in a human cervical explant infection model [53]. However, recent studies have shown that CV-N possesses cytotoxicity and mitogenic activity with pro-inflammatory responses in hPBMCs at nanomolar concentrations, which are very close to the protein's antiviral dose range [29], [30]. These deleterious and pro-infective actions have raised a serious concern for CV-N's candidacy toward microbicide use. Indeed, such activities are common features of lectins (with a few notable exceptions, e.g., GRFT [45]), necessitating careful examination of lectin-based HIV-1 inhibitors in these aspects. In spite of the similarity between CV-N and AH in sugar binding specificity, our results demonstrated that AH is devoid of cytotoxicity or mitogenic activity in hPBMCs at concentrations where the protein exerts potent anti-HIV-1 effects. We recognize that more thorough analyses, including inflammatory and immunogenicity endpoints, must be performed for higher doses of AH to conclude the protein's safety. Nevertheless, when combined with AH's exquisite specificity to Env glycans and preliminary results showing the lack of toxicity in a rabbit vaginal irritation assay with 0.1% AH solution (Tanaka et al. manuscript in preparation), our results provide a reasonable foundation supporting the protein's safety and should facilitate further investigation in this regard.

Microbicides will be required in massive amounts at low costs, considering the application frequency and estimated target population of those millions living in developing regions where HIV-1 infections are most prevalent. Accordingly, a key factor for the success of protein-based HIV-1 microbicides is the establishment of a scalable and cost-effective recombinant expression system; however conventional closed cell culture-based systems may not be able to meet these requirements [54]. Plants have recently emerged as an alternative recombinant expression system that has the potential to mass produce foreign proteins at substantially low costs [55], [56], [57]. In particular, the advent of recombinant plant virus vector systems enabled rapid and high-level expression of foreign proteins in plants. In the present study, we showed that rAH expressed via a TMV-based system can bind to gp120 and inhibit syncytia mediated by the Env-CD4 interaction, which demonstrates the protein's functional integrity. Although there is room for improvement (e.g., reducing rAH's aggregate formation *in planta*), we obtained a comparatively high expression level (up to 120 mg of *functional* rAH per kg of fresh leaf material) for plant-based expression systems in general [55]. In addition, the functional portion of rAH was readily separated and recovered from the aggregates after extraction (Fig. 5B). Consequently, we have successfully developed a novel rAH expression platform in plants. A logical and essential next step, which is currently underway, is the detailed characterizations of rAH in terms of molecular integrity, anti-HIV-1 efficacy, and safety using thoroughly purified material.

Compared to other lectins that have been reported to exhibit anti-HIV-1 effects (reviewed in [7]), AH's antiviral activity reported here is not the strongest in this class. Notwithstanding the moderate anti-HIV-1 efficacy, AH's apparent lack of mitogenicity and cytotoxicity in hPBMCs demonstrated here is a clear advantage of this protein over some of the other anti-HIV-1 lectins. Using rDNA technology, it may be possible to engineer rAH derivatives that have stronger anti-HIV activity while maintaining quiescence in the toxicological aspects. In fact, in our preliminary study, a recombinantly engineered AH dimer construct (native AH is a monomeric protein [12]) was shown to exhibit significantly stronger and broader anti-HIV-1 activity than the original monomer molecule. For example, two clade A viruses that showed strong resistance to AH (Q461.e2 and Q168.a2; see Table 1) were strongly neutralized by the dimer with IC_50_s less than 1 µg/ml (Tanaka & Matoba, manuscript in preparation). Based on the speed and robustness, the TMV-based rAH expression system will further facilitate the design of additional rAH analogues that have even more potent anti-HIV-1 activity and other desirable properties for microbicide use (e.g., producibility, bioavailability, formulability, etc.). Taken together, the TMV-based system may provide a viable means for the economical large-scale production as well as the design of rAH derivatives, which both significantly facilitate the development of rAH-based microbicide candidates.

In summary, we have demonstrated that: 1) AH exerts nanomolar-level antiviral activity against many clinically relevant HIV-1 strains of different clades and diverse cellular tropisms (with a possible exception for R5-type A clade viruses); 2) AH does not possess cytotoxicity or mitogenic activity in hPBMCs at doses showing broad anti-HIV-1 activity; and 3) the TMV-based system serves as a novel rAH expression platform that may facilitate molecular engineering and economical large-scale production. Based on these initial efficacy, safety, and producibility data, we propose that AH is promising as a precursor of new candidate HIV-1 microbicides targeting Env glycans. Further investigation on efficacy and safety, analysis of mechanisms of action, and design of potent analogues are warranted. Future studies should also investigate potential antiviral synergism between rAH derivatives and other antivirals toward an effective combination microbicide strategy.
